# Study on the treatment of postmenopausal osteoporosis with quercetin in Liuwei Dihuang Pill based on network pharmacology

**DOI:** 10.1186/s13018-022-03470-1

**Published:** 2023-01-09

**Authors:** Fuping Zhu, Wuping Li, Linhua Wang, Bing Dai, Zongyi Liu, Hang Wu, Ting Deng

**Affiliations:** 1 Department of Foot and Ankle Orthopedics, The First Affiliated Hospital of Hunan University of Traditional Chinese Medicine, Changsha, China; 2 Department of Extremities and Arthrosis, The First Affiliated Hospital of Hunan University of Traditional Chinese Medicine, Changsha, China; 3 Department of Pharmacy, The First Affiliated Hospital of Hunan University of Traditional Chinese Medicine, Changsha, China; 4 Clinical Nursing Teaching and Research Section, The Second Xiangya Hospital of Central South University, No. 139, Renmin Road, Furong District, Changsha, Hunan China

**Keywords:** Postmenopausal osteoporosis, Quercetin, PI3K/AKT signaling, PPI

## Abstract

**Background:**

Liuwei Dihuang Pill (LP) was verified to alleviate postmenopausal osteoporosis (PMOP) development. Nevertheless, the major constituent of LP and the related network pharmacology study remain unexplored.

**Methods:**

Protein–protein interaction was established to identify the downstream target of LP in PMOP, and the related signaling pathway was investigated by bioinformatics analysis. MC3T3-E1 cells were added to ferric ammonium citrate (FAC) to mimic osteoporosis in vitro. The osteoblasts were identified by Alizarin red staining. Western blot was applied to evaluate protein levels. In addition, Cell Counting Kit-8 (CCK8) assay was applied to assess cell viability, and cell apoptosis was assessed by flow cytometry.

**Results:**

Quercetin was the major constituent of LP. In addition, quercetin significantly reversed FAC-induced inhibition of osteogenic differentiation in MC3T3-E1 cells. In addition, quercetin notably abolished the FAC-induced upregulation of Bax, Caspase-3, FOS, JUN, TGFB1 and PPARD. In contrast, Bcl-2, p-mTOR/mTOR, p-AKT/AKT and p-PI3K/PI3K levels in MC3T3-E1 cells were reduced by FAC, which was restored by quercetin. Meanwhile, FAC notably inhibited the viability of MC3T3-E1 cells via inducing apoptosis, but this impact was abolished by quercetin. Furthermore, quercetin could reverse pcDNA3.1-FOS-mediated growth of FAC-treated osteoblasts by mediating PI3K/AKT/mTOR signaling.

**Conclusion:**

Quercetin alleviated the progression of PMOP via activation of PI3K/AKT/mTOR signaling. Hence, this study would shed novel insights into discovering new methods against PMOP.

**Supplementary Information:**

The online version contains supplementary material available at 10.1186/s13018-022-03470-1.

## Introduction

Postmenopausal osteoporosis (PMOP) is a subtype of systemic metabolic bone disease, in which downregulated estrogen levels are a major cause [1, 2]. As a result, postmenopausal women have a higher incidence of osteoporosis than premenopausal [3]. Osteoporosis easily leads to fragility fractures, and the morbidity has risen to 40–50% among women [4]. The imbalance between osteoclasts' resorption and osteoblast formation was known to cause the development of PMOP. This phenomenon might cause a declined bone mass, an increase in bone fracture and the modification of bone tissue structure [5]. Hence, it is urgent to explore novel strategies for treating PMOP.

Liuwei Dihuang Pill (LP) was known to be effective in nourishing the kidneys and attenuating tinnitus, dizziness and weakness. LP is composed of Poria cocos (Fuling), Paeonia suffruticosa (Mudanpi), wine dogwood meat (Jiuyurou), Chinese yam (Shanyao), dogwood (Shanzhuyu), Rehmannia glutinosa (Shudihuang) and Alisma (Zexie). In addition, LP is applied to increase male health and is used to alleviate kidney deficiencies. Previously, Xv et al. found some targets for treating PMOP with kidney-Yin deficiency through bioinformatics analysis, such as NCOA3, TCF4, DUSP6, PELI2, and STX7 [6]. Ge et al. showed that LP might treat kidney yin deficiency PMOP by upregulating CLCF1 gene expression and activating the JAK/STAT signaling pathway [7]. We hope to explore the active ingredients in LP for the treatment of PMOP. However, no studies deeply explored the treatment of PMOP with quercetin in LP. In recent studies, LP was suggested to have antiapoptotic [8] and antioxidative [9] impacts. This study decided to explore the major constituents of LP in PMOP. The cells were treated with quercetin, the main component of LP, to detect its effects on cell proliferation and apoptosis. The mechanism of quercetin in PMOP was also discussed.

With the progress of technology science, network pharmacology was known to be an important field [10, 11]. According to the principle of systems biology, network pharmacology is applied to elucidate the potential mechanisms underlying the function of drug therapy. Still, molecular docking is the systematic strategy to explore the association between large and small molecules [12, 13]. Our work decided to examine the potential mechanisms underlying the function of LP in PMOP by using network pharmacology. We hope our work will shed novel insights into the clinical value of LP.

## Material and methods

### Exploration of LP component targets

As mentioned above, the active constituents of LP were obtained: Fuling, Mudanpi, Jiuyurou, Shanyao, Shanzhuyu, Shudihuang and Zexie [14]. The ingredient screening conditions were oral bioavailability (OB) ≥ 30% and drug-like index (DL) ≥ 0.18. The prediction of targets was by TCMSP (https://tcmspw.com/tcmsp.php). And the non-human targets were removed after correction by the UniProt database (https://www.uniprot.org/).

### Exploration of targets in PMOP

With “PMOP” as the keyword, the related human genes were explored in GeneCards (http://www.genecards.org/) [15], National Center for Biotechnology Information (NCBI) gene (https://ncbi.nlm.nih.gov/) [16] and Online Mendelian Inheritance in Man (OMIM®) (http://omim.org) [17]. The median screening was performed in the GeneCards database according to the target scores for obtaining more relevant targets.

### Manufacture of Venn diagrams

The screened LP targets and PMOP targets were input into the Venn diagram creation software Venny 2.1, and 117 common targets were obtained.

### Protein–protein interaction (PPI) analysis

The above 117 common targets were entered into the STRING database (https://string-db.org/cgi/input.pl) to establish the PPI network, and the biological species was set as “Homo sapiens” and the confidence score > 0.7, the PPI network was obtained.

### Topological analysis

The PPI network was imported into Cytoscape 3.8.0, the NetworkAnalyzer performed topological analysis, and genes with a score greater than the average were selected as key targets by degree sorting. A total of 46 key targets were screened, and the top 20 were screened. The target points were drawn using R 4.0.3.

### Molecular complex detection (MCODE) cluster analysis

The PPI network was imported into Cytoscape 3.8.0, and the MCODE module was opened for gene cluster analysis and core target screening. A total of 6 gene clusters and 5 core genes were obtained.

### Gene Ontology (GO) analysis

Common targets of LP and PMOP were subjected to GO enrichment pathway analysis using R4.0.3. Referring to the STRING database, items with a corrected *P* value < 0.05 were screened. Bubble charts were drawn with the clusterProfiler package.

### Kyoto Encyclopedia of Genes and Genomes (KEGG)

Traditionally, KEGG pathway analysis was one strategy for discovering biological functions and pathways. KEGG analysis was applied using R 4.0.3. After installing and referencing the clusterProfiler package, bubble charts were drawn.

### Ultra-performance liquid chromatography-mass spectrometry (UPLC-MS) of LP

First, LP was dissolved in an extractant of 40% methanol + 40% acetonitrile + 20% H_2_O_2_. After standing for 10 min, it was sonicated at 4 °C. Let it stand for 1 h at -20 °C. Discard the pellet. The supernatant was drained with a freeze concentrator. The samples were redissolved in 50% acetonitrile + 50% H_2_O_2_. LP was analyzed using a Shimadzu Prominence UPLC system (Nexera UHPLC LC-30A, Kyoto, Japan) with QTRAP MS (SCIEX, QTRAP 5500). 3 μL sample was drawn and loaded onto a T3 column at 40 °C. The mobile phases comprised 0.1% aqueous formate (mobile phase A) and acetonitrile (mobile phase B). The flow rate of the mobile phase was set to 0.3 mL/min. Data were collected using Analyst 1.7.1 software (SCIEX), and relative amounts of quercetin were analyzed using MultiQuant 3.0.3 software (SCIEX).

### Preparation of LP medicated serum

Five male SD rats of 220–280 g (Hunan SJA Laboratory Animal Co., Ltd.) were acclimated for 1 week. Rats were gavaged 5.4 g/kg of LP twice daily for 5 days. Two hours after the last administration, the rats were anesthetized with 2% (w/v) pentobarbital (40 mg/kg), and the rats were killed.

### Cell culture and treatment

Mouse osteoblasts (MC3T3-E1) were bought from the ATCC (USA). In addition, cells were placed in Dulbecco's modified eagle medium (DMEM) (containing 10% FBS) with penicillin (100 U/mL, Gibco) at 37 °C in the condition of 5% CO_2_.

For establishing in vitro model of PMOP, cells were exposed to ferric ammonium citrate (FAC) (100 μmol/L, Sigma, USA) for 48 h.

To detect the function of quercetin in PMOP, cells were exposed to quercetin (Sigma, USA) (10/25/50 μM) for 48 h [18]. The cells in LP group were treated with 10% LP Medicated Serum for 48 h [19].

### Cell transfection

Osteoblasts (3 × 10^5^ cells/well) were cultured. When cells reached 70% confluence, they were transfected with pcDNA3.1-FOS (oe-FOS, Beyotime, China) or FOS siRNA (si-FOS, Beyotime, China) for 48 h using Lipofectamine 2000 (Invitrogen) in line with the protocol of the manufacturer.

### Alizarin red staining

The osteogenic differentiation in cells was assessed by alizarin red staining. Cells were seeded overnight. Polyformaldehyde (5%, 500 μL, Beyotime, China) and alizarin (200 μL, 30 min, Beyotime, China) were added to the medium, and the microscope was applied to observe the calcium nodules.

### Western blot detection

RIPA was applied to extract protein from cells. BCA was applied to assess the protein concentration. Subsequently, SDS-PAGE gel (10%) was applied to separate the proteins, and then proteins were transferred onto PVDF membranes. Primary antibodies were applied to incubate the membranes overnight after blocking for 1 h. Afterward, a secondary anti-rabbit antibody (SA00001-1; Proteintech, USA, 1:5000) was applied to incubate the membranes for 1 h. The primary antibodies were as follows: anti-Bax (ab32503, Abcam, Cambridge, MA, USA; 1:1000), anti-Caspase-3 (ab32351, Abcam; 1:5000), anti-Bcl-2 (ab59348, Abcam; 1:1000), anti-p-PI3K (ab182651, Abcam; 1:1000), anti-PI3K (ab227204, Abcam; 1:1000), anti-p-AKT (ab81283, Abcam; 1:5000), anti-AKT (ab108266, Abcam; 1:1000), anti-p-mTOR (ab109268, Abcam; 1:1000), anti-FOS (ab208942, Abcam; 1:1000), anti-JUN (66313-1-Ig, Proteintech; 1:1000), anti-TGFB1 (21898-1-AP, Proteintech; 1:1000), anti-PPARD (ab178866, Abcam; 1:1000), anti-mTOR (ab2732, Abcam; 1:2000) and anti-β-actin (66009-1-Ig, Proteintech; 1:5000). β-actin was applied for normalization.

### Cell Counting kit-8 (CCK8)

Cells (5 × 10^3^) were cultured overnight. Subsequently, cells were treated for 12, 24 or 48 h. Subsequently, cells were exposed to CCK8 (10 μl, Beyotime) for 2 h. A microplate reader was applied to assess the absorbance (450 nm).

### Flow cytometry

Cells were trypsinized, washed and re-suspended in binding buffer. Subsequently, APC-A (5 μl) and propidium (PI, 5 μl) were applied to stain the cells with no light for 15 min. Flow cytometer (BD, USA) was applied to calculate the cell apoptosis rate.

### Statistical analysis

SPSS v18.0 (SPSS, USA) was applied to analyze the data. Means ± SEM was applied to show the data. ANOVA (followed by the Tukey–Kramer post hoc test) was applied to analyze the differences among multiple groups, and independent samples t test was applied to analyze the differences between the two groups. *P* < 0.05 was considered significant.

## Results

### PPI network

**Fig. 1:**
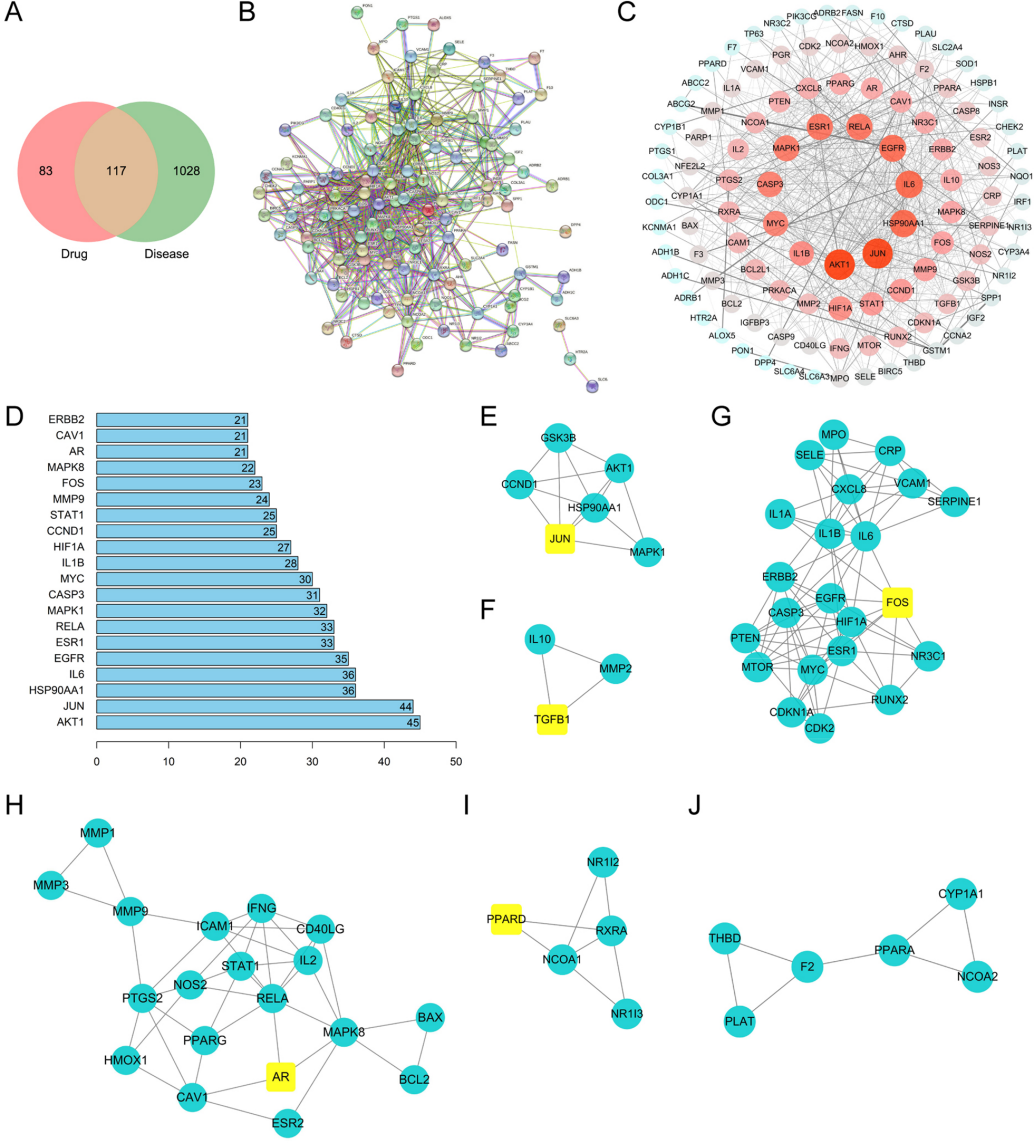
PPI network. **A** Input the screened targets and disease targets into the Venn diagram creation software and obtain 117 common targets, which were applied as the targets of drugs. The following pathway enrichment analysis was performed. **B**, **C** To construct the PPI network, the frequent targets and diseases were entered into the String. **D** Topological analysis shows the top 20 key targets. **E**–**J** In this study, the core genes were screened by MCODE analysis. Import the PPI network into Cytoscape 3.8.0, open the MCODE module for core target screening and gene cluster analysis

The screening conditions for active ingredients in LP were OB ≥ 30% and DL ≥ 0.18. They were corrected to remove non-human targets. 15 components and 23 targets were obtained from Fuling; 11 components and 156 targets were obtained from Mudanpi; 16 components and 72 targets were obtained from Shanyao; 20 components and 68 targets were obtained from Jiuyurou; 2 components and 30 targets were obtained from Shudihuang; 10 components and 5 targets were obtained from Zexie. After pooling and deduplication, 200 targets were obtained from LP. Using "PMOP" as the keyword, 1143 related genes were retrieved from the GeneCards database, 87 genes were obtained from the NCBI database, and 12 targets were obtained from the OMIM database. After the genes of these three databases were merged and deleted, 1145 genes related to postmenopausal osteoporosis were obtained. A total of 117 targets overlapped between LP and PMOP (Fig. 1A). The common targets of drugs and diseases were input into the String database to construct the PPI network. The biological species was set as "Homo sapiens", with a reliability > 0.7, and the PPI network was obtained. 117 nodes in this network, 719 edges, and the average degree value was 12.3 (Fig. 1B). Cytoscape software was used to draw a PPI network diagram, and its degree value was reflected according to the size and color of the circle. The larger the circle, the redder the color and the greater the degree value (Fig. 1C). Topological analysis was conducted by NetworkAnalyzer tool, and the top 20 genes were selected as key targets (Fig. 1D). After setting, the confidence score was retained, as illustrated in Fig. 1E–J. AKT1, JUN, HSP90A1, GSK3B, CCND1, AR, FOS, JUN, TGFB1, PPARD, etc., were PPI's core genes.

### The construction of constituent-disease target network

**Fig. 2:**
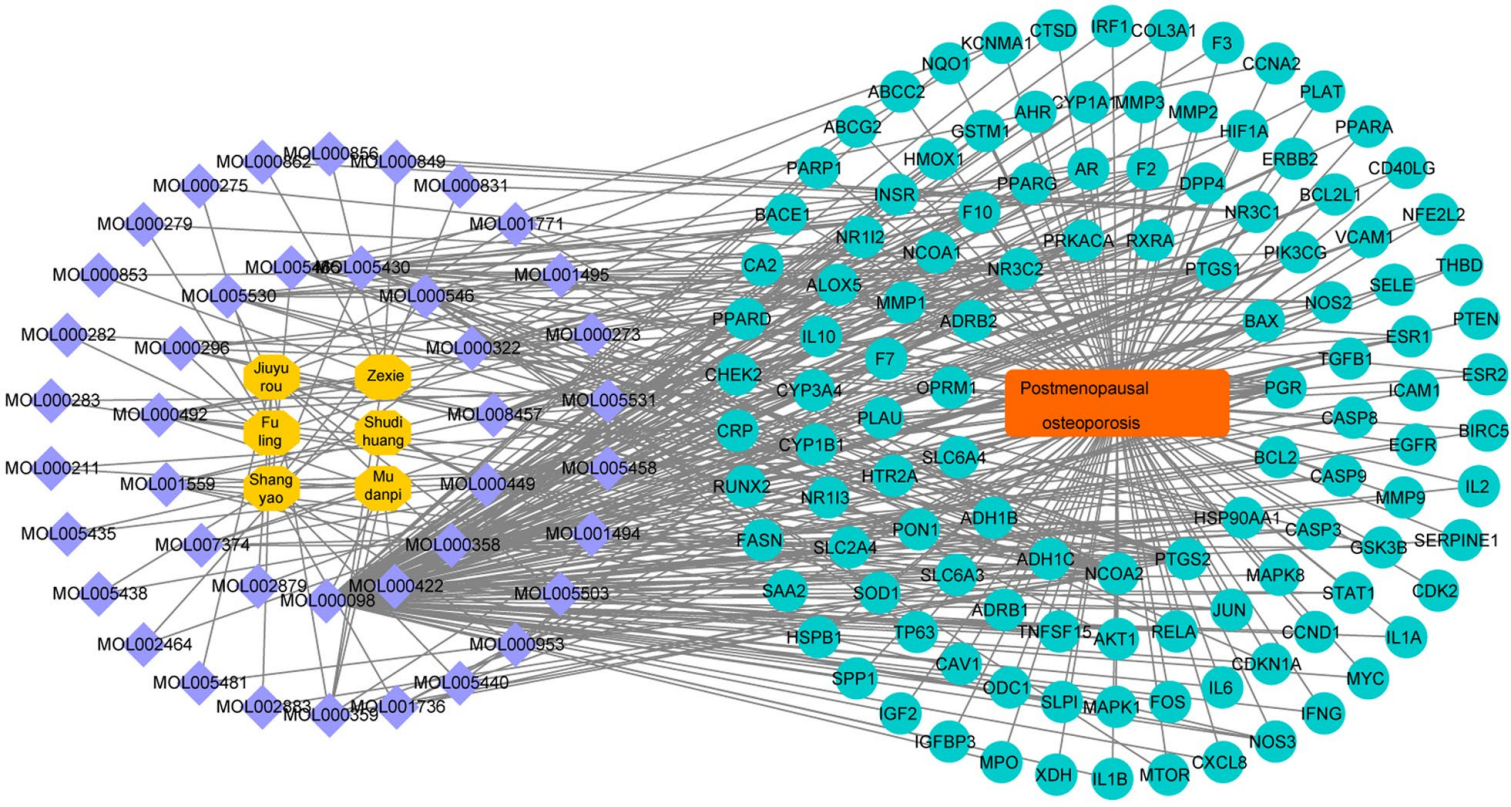
Construction of constituent-disease target network. Blue indicated the active constituent of LP, and green suggested the downstream targets of PMOP

To better understand the complex relationship between components, diseases and corresponding targets, we constructed a component-disease target network based on the included components, therapeutic diseases and targets. Components, diseases and targets were imported into Cytoscape 3.8.0 for drawing network diagrams (Fig. 2). Blue indicated the active constituent of LP (kaempferol, quercetin, stigmasterol, kadsurenone, beta-sitosterol, etc.). Meanwhile, BCL-2, PGR, RXRA, etc., were the downstream targets of PMOP.

### Screening of active constituents of LP

**Fig. 3:**
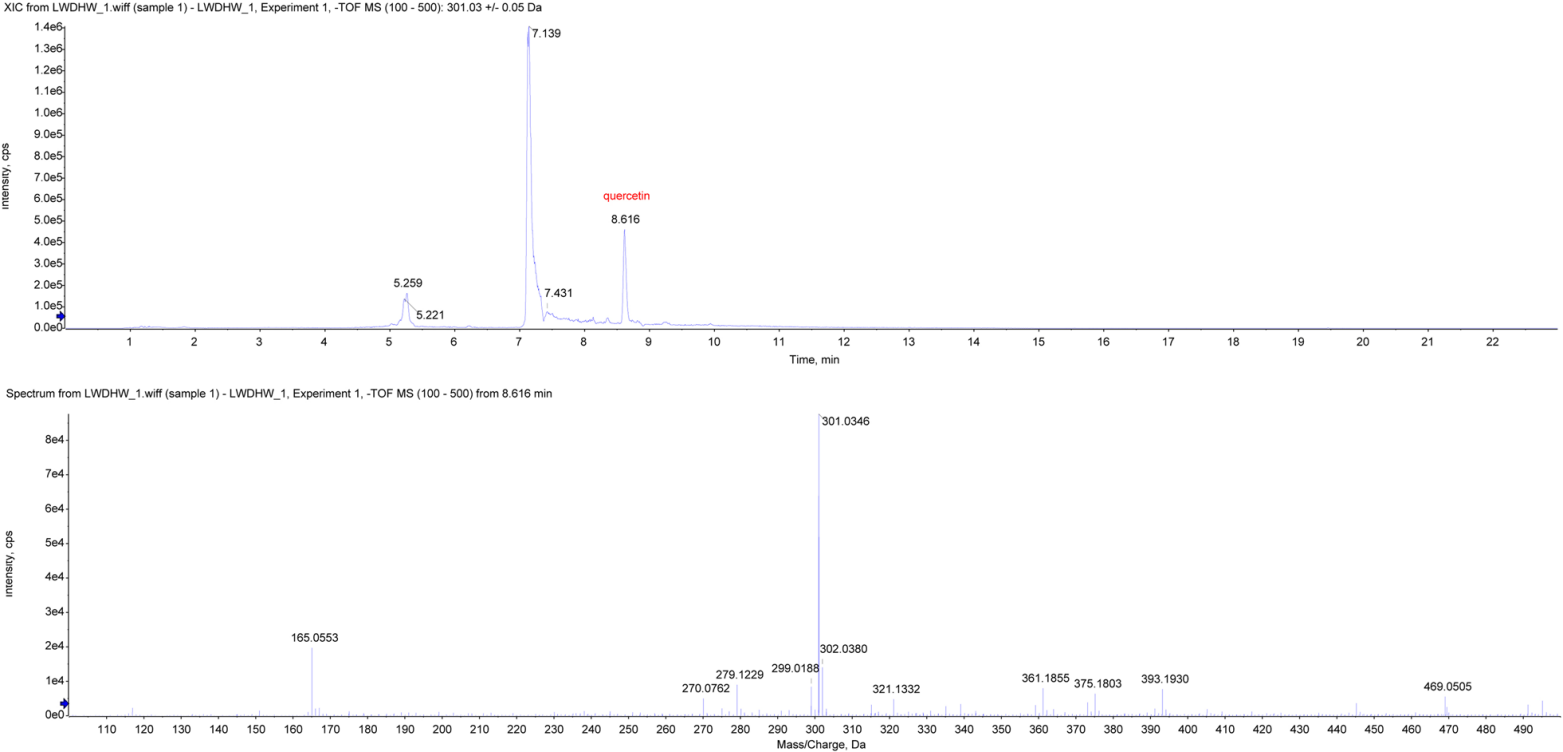
Screening of active constituents of LP. HPLC was used to identify the major active constituents of LP

Constituents of LP were ranked, and then detailed contents of active constituents are presented in Table 1. Quercetin, kaempferol, beta-sitosterol, stigmasterol and kadsurenone rank top five among the active constituents (Table 1). In addition, according to the data of HPLC, quercetin was confirmed to be the major constituent of LP (Fig. 3).

**Table 1:** Ingredient list of Liuwei Dihuang Pills

MOL ID	Name	Average shortest path length	Betweenness centrality	Closeness centrality	Degree
MOL000098	Quercetin	1.786585	0.21589	0.559727	91
MOL000422	Kaempferol	2.371951	0.047761	0.421594	40
MOL000358	Beta-sitosterol	2.591463	0.018425	0.385882	20
MOL000449	Stigmasterol	2.603659	0.025786	0.384075	16
MOL000322	Kadsurenone	2.70122	0.009648	0.370203	15
MOL008457	Tetrahydroalstonine	2.762195	0.007572	0.362031	15
MOL000546	Diosgenin	2.689024	0.012188	0.371882	14
MOL005430	Hancinone C	2.75	0.006617	0.363636	13
MOL005465	AIDS180907	2.77439	0.005688	0.36044	11
MOL005530	Hydroxygenkwanin	2.79878	0.003227	0.357298	9
MOL000296	Hederagenin	2.75	0.011265	0.363636	8
MOL000492	(+)-Catechin	2.810976	0.002344	0.355748	8
MOL000359	Sitosterol	2.689024	0.016158	0.371882	7
MOL001559	Piperlonguminine	2.945122	0.002184	0.339545	7
MOL001736	(-)-Taxifolin	2.884146	0.001197	0.346723	5
MOL007374	5-[[5-(4-methoxyphenyl)-2-furyl]methylene]barbituric acid	3.030488	6.93E−04	0.32998	5
MOL000953	CLR	2.810976	0.001446	0.355748	4
MOL001494	Mandenol	2.871951	7.86E−04	0.348195	4
MOL005440	Isofucosterol	2.810976	0.001446	0.355748	4
MOL005503	Cornudentanone	2.884146	8.36E−04	0.346723	4
MOL000273	(2R)-2-[(3S,5R,10S,13R,14R,16R,17R)-3,16-dihydroxy-4,4,10,13,14-pentamethyl-2,3,5,6,12,15,16,17-octahydro-1H-cyclopenta[a]phenanthren-17-yl]-6-methylhept-5-enoic acid	2.884146	0.002521	0.346723	3
MOL001495	Ethyl linolenate	2.896341	4.95E−04	0.345263	3
MOL001771	poriferast-5-en-3beta-ol	2.847561	8.18E−04	0.351178	3
MOL005458	Dioscoreside C_qt	2.847561	0.00216	0.351178	3
MOL005531	Telocinobufagin	2.908537	0.002028	0.343816	3
MOL000211	Mairin	3.018293	2.37E−04	0.331313	2
MOL000275	Trametenolic acid	3.067073	6.85E−04	0.326044	2
MOL000279	Cerevisterol	3.067073	6.85E−04	0.326044	2
MOL000282	Ergosta-7,22E-dien-3beta-ol	3.018293	8.90E−04	0.331313	2
MOL000283	Ergosterol peroxide	3.018293	8.90E−04	0.331313	2
MOL000831	Alisol B monoacetate	3.128049	7.08E−04	0.319688	2
MOL000849	16β-methoxyalisol B monoacetate	3.128049	7.08E−04	0.319688	2
MOL000853	Alisol B	3.042683	0.001395	0.328657	2
MOL000856	Alisol C monoacetate	3.128049	7.08E−04	0.319688	2
MOL000862	[(1S,3R)-1-[(2R)-3,3-dimethyloxiran-2-yl]-3-[(5R,8S,9S,10S,11S,14R)-11-hydroxy-4,4,8,10,14-pentamethyl-3-oxo-1,2,5,6,7,9,11,12,15,16-decahydrocyclopenta[a]phenanthren-17-yl]butyl] acetate	3.128049	7.08E−04	0.319688	2
MOL002464	1-Monolinolein	2.993902	0.002389	0.334012	2
MOL002879	Diop	3.042683	1.90E−04	0.328657	2
MOL002883	Ethyl oleate (NF)	2.932927	2.06E−04	0.340956	2
MOL005435	24-Methylcholest-5-enyl-3belta-O-glucopyranoside_qt	2.981707	1.98E−04	0.335378	2
MOL005438	Campesterol	2.981707	1.98E−04	0.335378	2
MOL005481	2,6,10,14,18-Pentamethylicosa-2,6,10,14,18-pentaene	2.957317	1.97E−04	0.338144	2

### GO enrichment and KEGG analysis

**Fig. 4:**
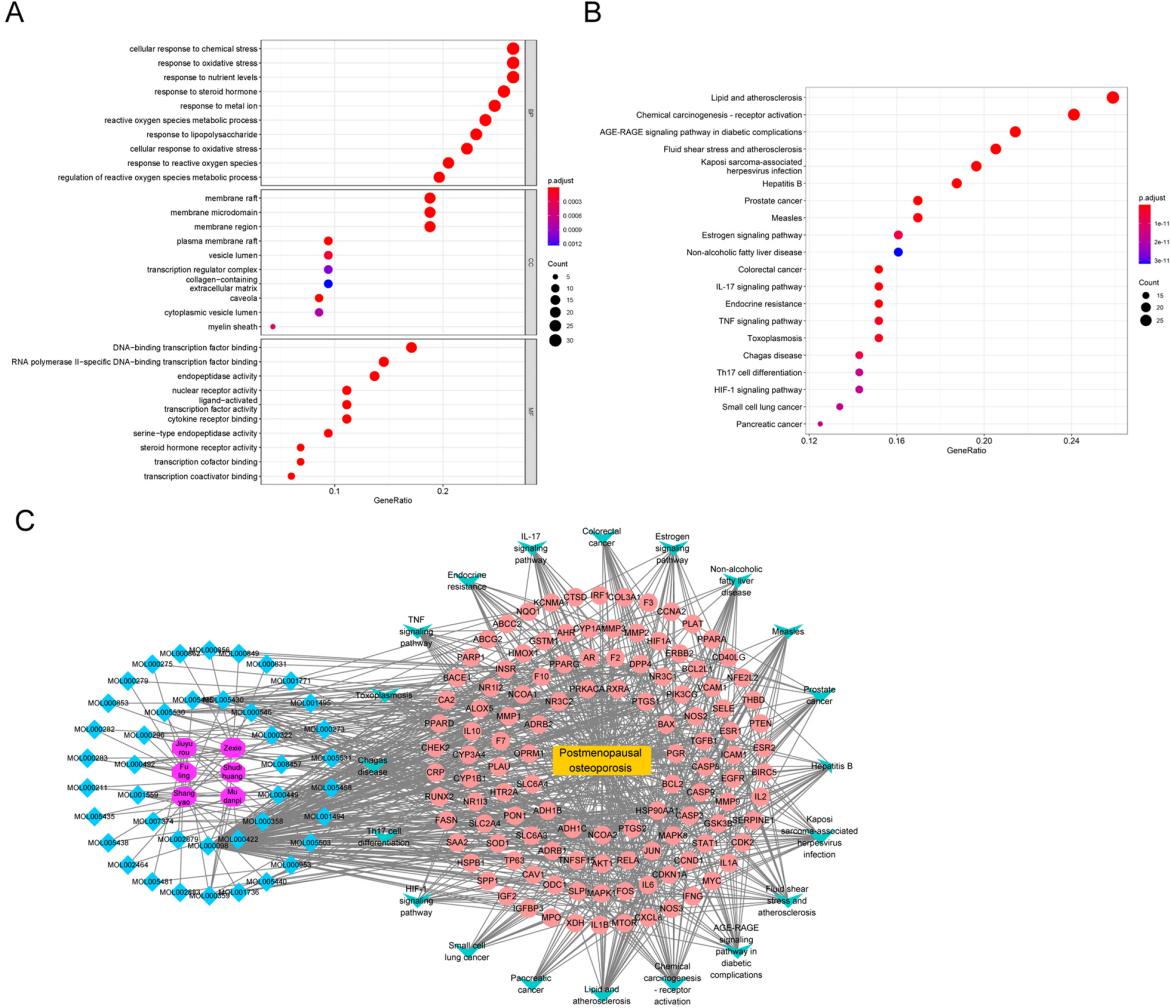
GO enrichment and KEGG analysis. **A** The procedure of GO enrichment analysis was performed by applying the clusters with high scores (top 3). **B** ClusterProfiler package of R language was applied for establishing KEGG pathway analysis. **C** The constituent-disease pathway target network was constructed

Targets common to LP and PMOP were enriched by GO, including biological process (biological process, BP), molecular function (molecular function, MF), and cellular component (cell component, CC). Citing the String database, the items with a corrected *P* value < 0.05 were screened (Fig. 4A). The data revealed that the most common biological process among downstream targets of PMOP was a cellular response to chemical stress; the most enriched cellular component was membrane raft, and the most enriched molecular function was DNA-binding transcription factor binding. In addition, KEGG pathway analysis was conducted with the ClusterProfiler package of R language. They were sorted by adjusting the *P* value, and the terms ranked top 30 are listed in Fig. 4B. The results suggested the downstream targets of PMOP were closely associated with lipid and atherosclerosis. Meanwhile, the constituent-disease pathway target network is presented in Fig. 4C. Blue indicated the active constituent of LP. In addition, BAX, ADH1C, SLC6A1, etc., were found to be the downstream targets of PMOP and LP. The blue was the compound, the pink was the target of the traditional Chinese medicine on the disease, the green was the most significant top 20 pathways, the yellow was the disease, and the purple was the traditional Chinese medicine.

### Quercetin significantly reversed FAC-induced apoptosis in osteoblasts via activating PI3K/AKT/mTOR

**Fig. 5:**
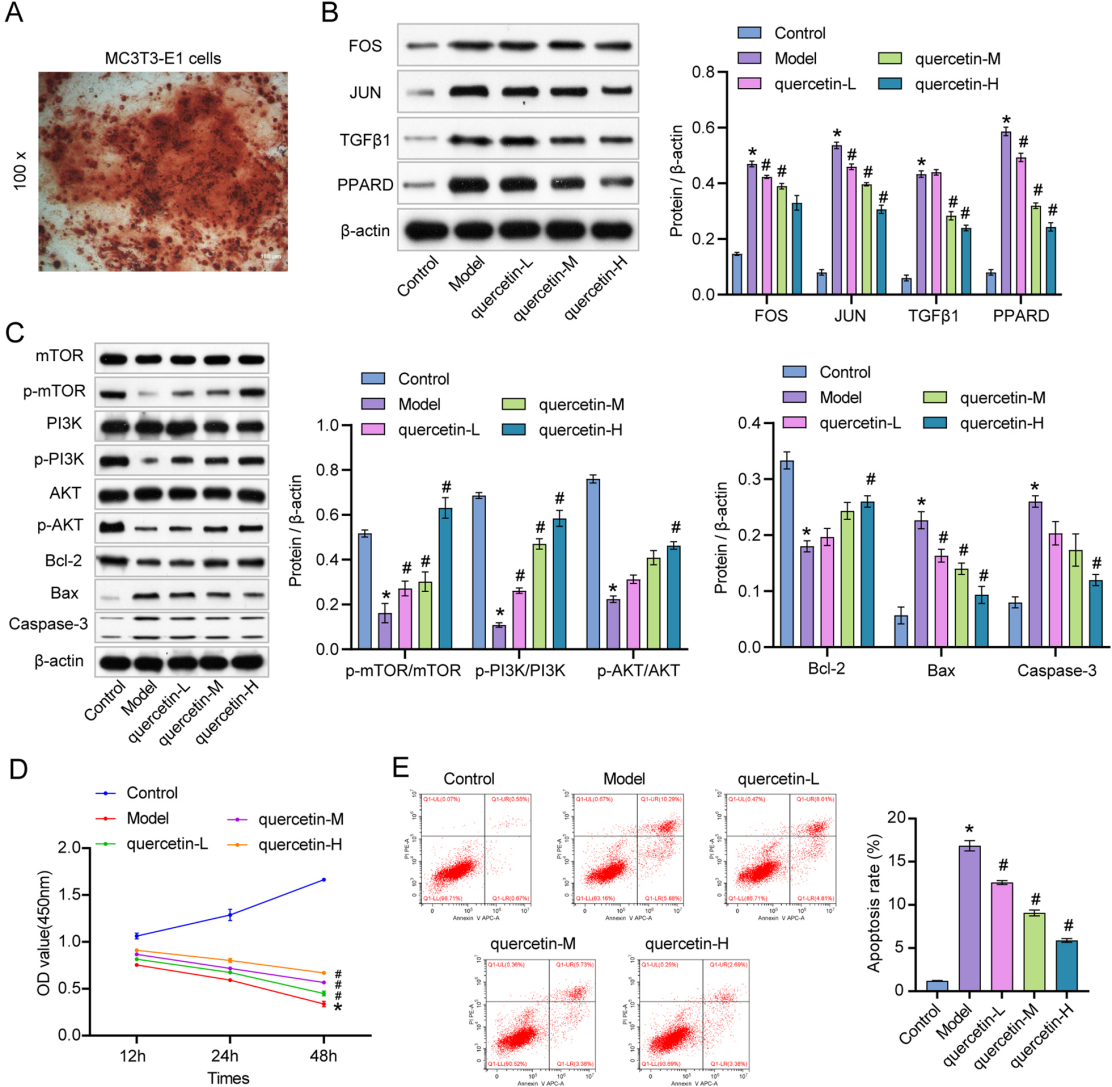
Quercetin significantly reversed FAC-induced apoptosis in osteoblasts by activating PI3K/AKT/mTOR. **A** MC3T3-E1 cells were identified by Alizarin red staining. The scale bar: 100 μm. **B** MC3T3-E1 cells were exposed to FAC, FAC + quercetin (L), FAC + quercetin (M) or FAC + quercetin (H). The expressions of FOS, JUN, TGF-β1, and PPARD in MC3T3-E1 cells were investigated by western blot. **C** The expressions of p-mTOR, mTOR, p-PI3K, PI3K, p-AKT, AKT, Bcl-2, Bax, and Caspase-3 in MC3T3-E1 cells were assessed by western blot. **D** The viability of MC3T3-E1 cells was assessed by CCK8 assay. **E** MC3T3-E1 cell apoptosis was assessed by flow cytometry. *P < 0.05 compared to control. ^#^*P* < 0.05 compared to model

Alizarin red staining was performed to identify the osteoblasts. As indicated in Fig. 5A, significant calcium deposits were observed, suggesting that cells were osteoblasts. We treated MC3T3-E1 cells with different doses of FAC to screen out the most suitable FAC concentration. We found that cells were exactly half lethal when treated with 100 μmol/L FAC. Moreover, 100 μmol/L FAC also affected cell apoptosis to some extent (Additional file 1: Figure S1). Therefore, we chose 100 μmol/L FAC to continue the subsequent experiments. The result showed that FAC significantly upregulated the levels of FOS, JUN, TGF-β1 and PPARD in MC3T3-E1 cells, which was dose-dependently reversed by quercetin (Fig. 5B). Consistently, FAC-induced upregulation of Bax and Caspase-3 was dose-dependently abolished by quercetin (Fig. 5C, D). In contrast, the levels of PI3K, p-PI3K, p-mTOR, AKT, p-AKT, mTOR and Bcl-2 in MC3T3-E1 cells were notably inhibited by FAC, which was rescued by quercetin (Fig. 5C, D). Meanwhile, FAC significantly decreased MC3T3-E1 cell viability, but the inhibitory impact of FAC was attenuated by quercetin (Fig. 5E). Furthermore, the apoptotic impact of FAC was also reversed by quercetin (Fig. 5F). Thus, quercetin significantly reversed FAC-induced apoptosis in osteoblasts via activating PI3K/AKT/mTOR. To show the efficacy of Liuwei Dihuang Pills, we added the Liuwei Dihuang Pills group for comparison. The results of CCK8 and flow cytometry showed that the therapeutic effect of Liuwei Dihuang Wan was similar to that of middle-dose quercetin, and its therapeutic effect was significantly lower than that of high-dose quercetin (Additional file 2: Figure S2).

### Quercetin significantly regulated the proliferation of osteoblasts through the mediation of FOS

**Fig. 6:**
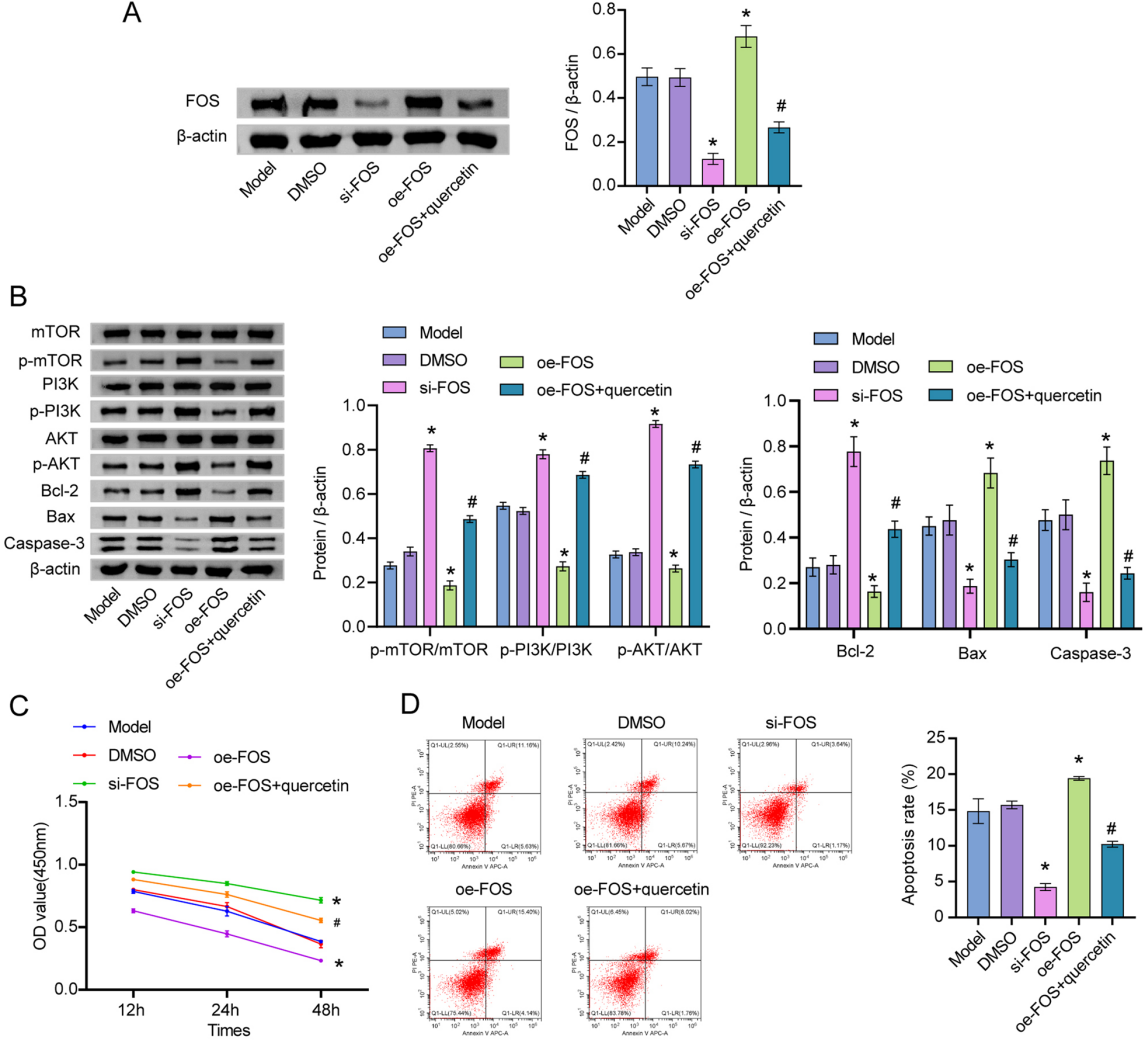
Quercetin significantly regulated the proliferation of osteoblasts through mediation of FOS. Osteoblasts were exposed to FAC, FAC + DMSO, FAC + si-FOS, FAC + oe-FOS or FAC + oe-FOS + quercetin. **A** The protein level of FOS in osteoblasts was assessed by western blot. **B** The expressions of p-mTOR, PI3K, AKT, p-AKT, p-PI3K, mTOR, Bax, Bcl-2 and Caspase-3 in osteoblasts were tested by western blot. **C** The viability of osteoblasts was assessed by CCK8 assay. **D** The apoptosis of osteoblasts was assessed by flow cytometry. *P < 0.05 compared to model. ^#^*P* < 0.05 compared to oe-FOS

The western blot was performed further to explore the relationship between quercetin and FOS in osteoblasts. As shown in Fig. 6A, FOS siRNA significantly inhibited the level of FOS in FAC-treated osteoblasts, while pcDNA3.1-FOS exerted the opposite effect. The effect of pcDNA3.1-FOS on FOS level was notably reversed by quercetin (Fig. 6A). In addition, the levels of p-mTOR, p-PI3K, p-AKT and Bcl-2 in FAC-treated osteoblasts were upregulated by FOS silencing but inhibited by pcDNA3.1-FOS. In contrast, the expressions of Bax and caspase-3 exhibited the opposite trend (Fig. 6B). Moreover, the effect of FOS overexpression on these proteins was restored by quercetin (Fig. 6B). Meanwhile, FOS knockdown could increase the viability and inhibit the apoptosis of FAC-treated osteoblasts; however, upregulation of FOS exhibited the opposite effect (Fig. 6C, D). Furthermore, pcDNA3.1-FOS-mediated osteoblast growth was reversed by quercetin (Fig. 6C, D). In summary, quercetin significantly regulated the proliferation of osteoblasts through the mediation of FOS.

## Discussion

According to Bayesian network meta-analysis, we found that corticosteroids, denosumab, Ibandronate, etc., can be effective drugs for the treatment of osteoporosis [20, 21]. However, these drugs have their limitations, leading to secondary osteoporosis, etc. Recent studies have shown that biochemical markers of bone turnover (BTM), such as bone alkaline phosphatase, can be used to monitor drug therapy for osteoporosis [22, 23]. However, the treatment of PMOP remains a great challenge clinically. Traditional Chinese medicines were verified to attenuate multiple disease development (including inflammation) [24, 25]. In this study, LP was found to alleviate PMOP progression. Additionally, quercetin was verified to be the major active constituent of LP. Hence, this work first discovered the impact of LP and quercetin in PMOP and confirmed that quercetin might be considered a novel PMOP treatment agent.

This work discovered the mechanisms of LP on PMOP, the major constituents and downstream targets. LP constituent-target network revealed the foundation of LP. Flavonoids are the majority of the major constituents of LP. Besides, quercetin is a polyphenolic flavonoid that exerts anti-tumor activity, especially in traditional Chinese medicine [26, 27]. In vitro studies have paid attention to chemopreventive activity and mechanisms underlying the function of quercetin in PMOP.

Recent reports suggested that PI3K/AKT/mTOR pathway plays a vital role in the cellular process [28, 29]. The PI3K/AKT/mTOR pathway is closely related to cell proliferation and apoptosis [30, 31]. In addition, p-AKT and p-PI3K are generally downregulated when osteoporosis occurs, illustrating that the progression of osteoporosis might be associated with cell injury [29, 32, 33]. This work firstly assessed the relation between PI3K/AKT/mTOR and quercetin in PMOP, confirming that quercetin restored FAC-induced cell injury during PMOP through activation of PI3K/AKT/mTOR signaling.

JUN, FOS and TGF-β are key modulators in pyroptosis [34, 35]. Meanwhile, these three proteins were also upregulated during the progression of osteoporosis [36–38]. Consistently, our work demonstrated that quercetin could abolish FAC-induced cell injury via the downregulation of JUN, FOS and TGF-β.

Previous studies have shown that inhibition of FOS in Raw264.7 cells can inhibit RANKL-induced osteoclastogenesis, which may be an effective way to treat osteoporosis [39]. In addition, studies have shown that garcinol can reduce RANKL-induced osteoclastogenesis by regulating the PI3K/AKT/mTOR signaling pathway and the downstream factor c-FOS [40]. Cnidium lactone regulated osteoporosis by regulating c-FOS/NFATc1 signaling pathways through p38 MAPK and PI3K-Akt [41]. Further, we investigated its effect on MC3T3-E1 cells by overexpressing FOS. Quercetin significantly modulates osteoblast proliferation via FOS mediation.

Some shortcomings existed in this work: the mechanisms underlying the impact of quercetin in PMOP remain further discovered. Thus, more detections are essential in the future.

In summary, quercetin alleviated PMOP progression via activation of PI3K/AKT/mTOR. Thus, quercetin might act as a crucial agent for PMOP treatment.

## Supplementary Information

**Additional file 1: Figure S1**. Screening for quercetin concentrations. MC3T3-E1 cells treated with different concentrations of FAC (0, 20, 50, 100, 200 μmol/L). (A) CCK8 assay was used to detect the activity of MC3T3-E1 cells. (B) MC3T3-E1 cell apoptosis was assessed by flow cytometry. *P< 0.05 compared to 0 μmol/L FAC.

**Additional file 2: Figure S2**. The effects of LP and quercetin on cell viability and apoptosis. (A) The viability of MC3T3-E1 cells was assessed by CCK8 assay. (B) MC3T3-E1 cell apoptosis was assessed by flow cytometry. *P< 0.05 compared to control. #P< 0.05 compared to model.

## Data Availability

The data used to support the findings of this study are included in the article.
