# Supplementary figures and images for: Study on the treatment of postmenopausal osteoporosis with quercetin in Liuwei Dihuang Pill based on network pharmacology
Source: J Orthop Surg Res. 2023 Jan 9;18:21. doi: 10.1186/s13018-022-03470-1 (PMC9827666; doi:10.1186/s13018-022-03470-1)

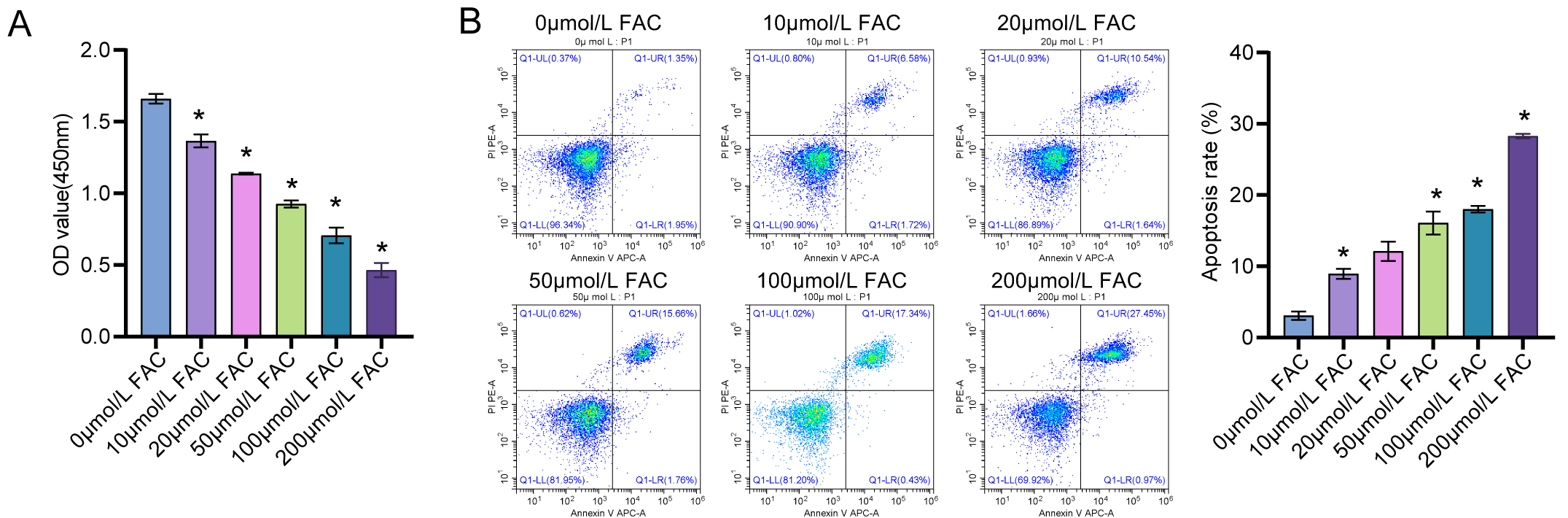

Supplement: Supplementary file 1 — Additional file 1: Figure S1. Screening for quercetin concentrations. MC3T3-E1 cells treated with different concentrations of FAC (0, 20, 50, 100, 200 μmol/L). (A) CCK8 assay was used to detect the activity of MC3T3-E1 cells. (B) MC3T3-E1 cell apoptosis was assessed by flow cytometry. *P< 0.05 compared to 0 μmol/L FAC. [file 13018_2022_3470_MOESM1_ESM.jpg]

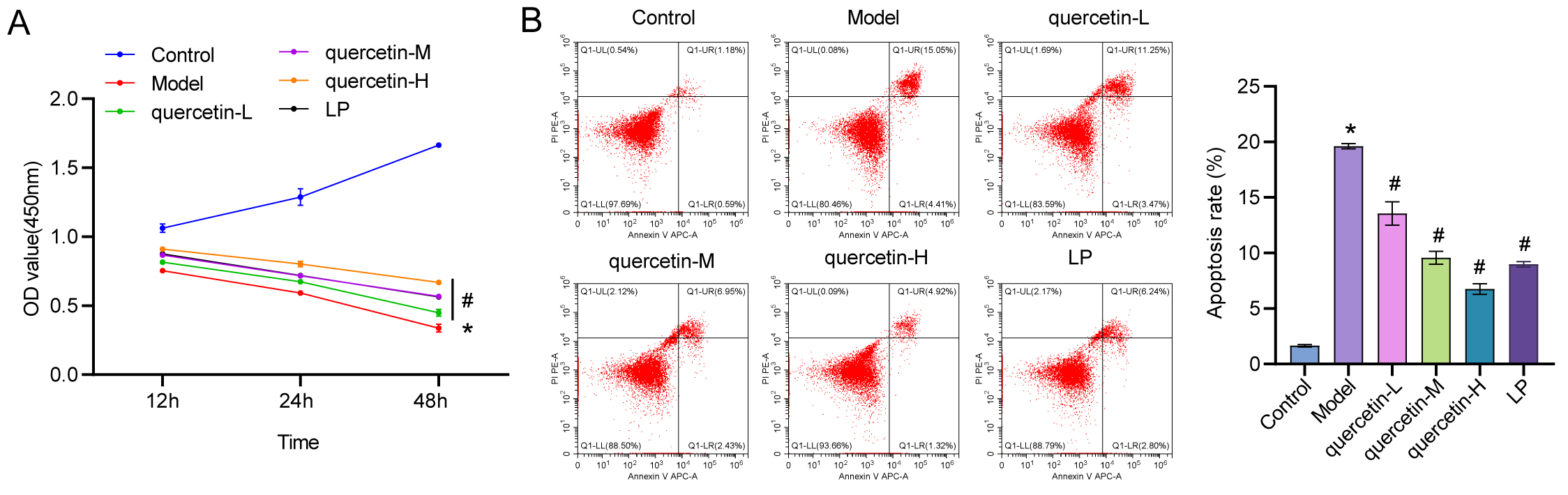

Supplement: Supplementary file 2 — Additional file 2: Figure S2. The effects of LP and quercetin on cell viability and apoptosis. (A) The viability of MC3T3-E1 cells was assessed by CCK8 assay. (B) MC3T3-E1 cell apoptosis was assessed by flow cytometry. *P< 0.05 compared to control. #P< 0.05 compared to model. [file 13018_2022_3470_MOESM2_ESM.jpg]
